# Supplementary material for: Commissioning a newly developed treatment planning system, VQA Plan, for fast-raster scanning of carbon-ion beams
Source: PLoS One. 2022 May 10;17(5):e0268087. doi: 10.1371/journal.pone.0268087 (PMC9089877; doi:10.1371/journal.pone.0268087)
Supplement: Attachment — Submitted filename: Review comments of PONE-D-21-22235_20211027.docx [file pone.0268087.s003.docx]

**Review comments of PONE-D-21-22235**

**Commissioning a newly developed treatment planning system, VQA Plan, for fast raster scanning of carbon-ion beams**

**General comments:**

The readability is acceptable in general with minimal grammar errors. It does come across a little dense at times. Although the manuscript is reasonably well readable, the English could be improved.

1. Paper structure and presentation are too lengthy in the current version and need to be more concise.
2. Tables and figures, especially the quality of all figures, need to be improved in the revision.
3. Language also needs to be improved by native English speakers. Please consult the reviewers' comments for more information.

The manuscript is lacking of the core value introduction of VQA which is a flaw that detracts from the positive contributions of this article. The author is encouraged to address more superiority of their planning system VQA.

Specific comments:

Line 48 accuracy for heterogeneous media

The singular noun **accuracy** follows a number other than *one*. Consider changing the noun to the plural form. Correct to: accuracies

Line111 optimized for each individual

**Each individual** may be redundant.

Line114 gland (HSG) tumor cells has been

The singular verb **has** does not appear to agree with the plural subject **cells**. Consider changing the verb form for subject-verb agreement.

Line 118 beams, and the response to individual tumors is considered on the basis of the…

It appears that you have an unnecessary comma in a compound subject. Consider removing it.

The verb **is** does not seem to agree with the subject. Consider changing the verb form. Correction: Is to are

The phrase **on the basis of** may be wordy. Consider changing the wording. Correction: Based on

Line 130 provide 100 different energy selections between 73.3 and 430.0 MeV/u from 12 accelerated energies,

Please clarify 100 different energies and 12 accelerated energies, what is the difference between these two energies.

Line 137 The main dose monitor checks the dose remaining at the given position, and a spot position monitor (SPM) confirms the position of the spot.

Does this specify for one carbon energy? or for every energy? Please add more explanations.

Line 138 position, and a spot position monitor (SPM) confirms the position of the spot. A monitor

It appears that you have an unnecessary comma in a compound subject. Consider removing it.

Line 139 unit (MU) is defined on the basis of a fixed amount of charge collected in the ionization

The phrase **on the basis of** may be wordy. Consider changing the wording. Correction: Based on

Line 140 chamber of the main dose monitor, and it corresponds to a physical dose to water of

Correction: the water

Line 153 The input data required for the scanned carbon-ion beam are integral depth doses (IDDs), the in-air lateral profiles, measurements relating to fragmentation, and the absolute dose and absolute dose correction factor.

Please add more explanations on what type of ion chamber you used for (IDDs), in-air lateral profiles as well as the data with a range shifter (RS) measurements.

Line163 measurement data are described in [2], so only a short summary

**Short summary** may be redundant. Correction: removed

Line 173 , (1)

It appears that you have improperly spaced some punctuation. Consider removing a space.

Line 180 first component (*n* = 1) represents the incident carbon-ion beam,

It appears that you have an unnecessary comma in a compound object. Consider removing it.

Line 199 multiplying the beam current defined at every control point by the time period.

**Time period** may be redundant. Keep only one word.

Line 200-203 Please address how to deal with the transit dose from one spot to the other spot in Intensive Modulated Carbon Therapy of your raster scanning facility.

Line 203 of the dose needed to calculate the beam width in the SPM is less than 0.00018 MU

What is the precise control mechanism for such a low MU?

Line 204 5) if the ratio of the stopping dose and the moving dose is not 1:1.

Please define what are: stopping dose and the moving dose?

Line 212 photons and for carbon ions using a specified survival level (10%)

Please clarify what is the specified survival level (10%)? Is that HSG? Following the same question, we all realize there are only one normal issue cell line (such as HSG) adopted by using a cell survival model to predict the carbon ion RBE, did your institute ever try to use individual tumor cell line for predicting the corresponding RBE to achieve disease by disease precision treatment in carbon ion beam treatment? Despite the difficult to do individual tumor cell line for predicting the RBE to achieve disease by disease precision treatment, what are your comments for this strategy?

Line 214 , (3)

It appears that you have improperly spaced some punctuation. Consider removing a space.

Line 225 , (5)

It appears that you have improperly spaced some punctuation. Consider removing a space.

Line 226 , (6)

It appears that you have improperly spaced some punctuation. Consider removing a space.

Line 237 contributions from the carbon ions and from

It appears that the preposition **from** is redundant. Consider removing it.

Line 238 Gaussian form, the first component (*n* = 1) represents the carbon isotopes,

It appears that you have an unnecessary comma in a compound object. Consider removing it.

Line 254 where *dclin,i* denotes the clinical dose at voxel *i*. The scaling factor 1.46

It seems that preposition use may be incorrect here. It is to be: of 1.46.

The author declares the scaling factor 1.46 was determined by connecting the modeled RBE to the clinical experience with neutron therapy in the report of Biophysical characteristics of HIMAC clinical irradiation system for heavy-ion radiation therapy, please explain or upon your opinion why use neutron to define carbon heavy ion scaling factor since neutron is neutral particle while carbon ion used for radiation therapy is charged particle.

Line 264 parameters needed

It seems that you are missing a verb. Consider adding it. It is to be: are needed

Line 270 of the absolute doses to convert the IDDs to absolute values.

Please describe how you define the absolute doses? Where to do the measurement along the IDDs in different carbon ion energies?

Line 270 of the absolute doses to convert the IDDs to absolute values. The absolute correction factor is used to compensate for the difference between the calculated physical absolute dose and that obtained from the measurements.

What is the absolute correction factor? Please clarify the definition and base on what to compensate the difference of physical absolute dose between calculation and measurements?

Line 274 **Determination of the CT-number-to-relative-stopping-power curve, validation,** **and range error estimation**

In this section, what is the abbreviation of RSP? Please provide your methods and experiences on how to deal with a patient with artifacts occurred by I-125 seeds implant, or by other metal materials which the CT number exceeds your CT-number-to-RSP calibration table, and the dose calculation accuracy under these artifacts. For those patients who have metal materials inside or beside the lesion, will you gona treat by heavy ion? If yes, what is your dose confidence? If not for treatment, what is your next strategy for patients?

In **TPS validation** section in line 359 We created all the fields by superimposing pencil beams. We obtained measurements with the Advanced Markus chamber, except that we used the PinPoint chamber for field sizes less than 40 mm × 40 mm

According to the author’s description, for all clinical data implemented to your VQA TP system were measured by Advanced Markus chamber except the use of PinPoint chamber for field sizes less than 40 mm × 40 mm. The reviewer would like to ask how did you do the effective point of measurement correction of these two chambers?

Line 289 eV [21], and the particle velocity relative to the speed of light in vacuum

The noun phrase **vacuum** seems to be missing a determiner before it. Consider adding an article.

Correction: in a vacuum

Line 305 the relationship between CT number and RSP by means of

The phrase **by means of** may be wordy. Consider changing the wording.

Consider to use: using, utilizing, employing, through

Line 307 We conducted an experiment

The phrase **conducted an experiment** may be unnecessarily wordy. Consider replacing the noun with a corresponding verb.

Consider to use: experimented

Line 318 method can be found in [25, 26]. In short, uncertainties in patient

Correct to: in patient’s

Line 333 mm, using the StingRay

Please clarify the term StingRay.

Line 335 IDDs on the dose–area product (DAP) [27] obtained

Correct to: were obtained

Line 372 The calculation grid is fixed at 2.0 mm × 2.0 mm × 2.0 mm and is not adjustable by the user.

What if you have to treat a small lesion less than 2 mm like an eye ball melanoma then you restrict the planner changing the resolution for better dosimetric evaluation inside the lesion and the surrounding normal critical tissues/organs?

Line 375 **Validation in heterogeneous media**

The author declares to place lung- and bone-equivalent materials 40 mm upstream from the surface of the water phantom to investigate the heterogeneous media, well, it seems at best to investigate the attenuation of the materials interested, please try to use Gafchromic film or of your own way for the measurement of dose profile inside the heterogeneous materials, especially the detail measurement of beam profile at the entrance and exit dose along the heterogeneous material.

Line 387 We measured the RSP for each material comprising the phantom in the same manner as in the experiment used to validate the CT-number-to-RSP curve, and this was reflected in the calculation by overwriting the CT-number function after inversely estimating the CT number of the measured RSP in the created

CT-number-to-RSP table because the material comprising the phantom was not real tissue and the CT number acquired from the CT image was not as accurate as that of the tissue.

Please re-write these sentences item by item to avoid confused. At this moment, reader couldn’t catch what you try to express.

Line 377 150 mm × 150 mm × 20 mm of acrylic and 30 mm × 30 mm × 20 mm of lung-

Correct to: of the lung-

Line 378 bone-equivalent materials was

Correct to: were

Line 382 point doses along the central axis and measure

Consider changing the verb form for subject-verb agreement.

Correct to: measures

Line 392 tissue and the CT number acquired from the CT image was not as accurate as that

Correct to: as of that

Line 399 TPS using the RBE measurements reported in [2],

Please add Reference in front of [2], and correct line 844 Therapy Center. J Appl Clin Med Phys. Forthcoming. Forthcoming to: Article-in-press

Line 442 from six head and neck cancer cases treated in the OHITC. In the prostate

Correct to: In prostate

Line 446 **Results**

All figures are in very poor image qualities. All figures should be retouched and gallery proofed in the next version.

Line 448 Fig 1 (a) shows the determined CT-number-to-RSP curve. The solid line is for small-diameter cases, such as the head and neck region, and the dashed line is for large-diameter cases, such as the abdominal region.

Please explain the reason what cause the RSP of the body lower than that of the head at a higher CT numbers?

Line 467 Figs 1 (d) and (e) the results of our experimental verifications.

Add showed after (e)

Line 469 differences between the RSP determined from the calibrated curves and from

It appears that the preposition **from** is redundant. Consider removing it.

Line 472 tissue,

It appears that you have an unnecessary comma in a compound subject. Consider removing it.

Line 503 **Fig 3. Comparison of IDDs from the TPS calculations and from**

It appears that the preposition **from** is redundant. Consider removing it.

**Fig 3. Comparison of IDDs from the TPS calculations and from measurement for all 100 energies.** The circles show the measured IDDs and the solid lines represent the calculated IDDs. The lower graph shows the differences in R90 in units of millimeters for all energies.

The author should present no more than 10 IDDs between the smallest and the largest carbon ion energies to avoid the crowded and fussy noise in Fig. 3.

Line 512 **Fig 4. Lateral dose profiles in water at selected energies and different positions.**

It is difficult to read each figure’s legendary, please correct. Please show Fig. 4 each profile’s tails both at the right and left side to a reasonable margin. Besides, what if the scanning beam passes over the desired margin, is there any erroneous proof mechanism to avoid scanning beam out of control?

Line 522 **Fig 5. Comparison of the absolute doses from the TPS and from**

It appears that the preposition **from** is redundant. Consider removing it.

What is the corresponding RBE in the same LET but different fraction size in your VQA TPS? Please tabulate the results for representative carbon ion energies. For example, please make the comparison for different OARs(skin, Spinal cord, kidney, liver after partial heaptectomy, Intestine, et al) with the endpoint by different fractions in the fix LET to get the RBE ( like as a, b, c, d are the desired items shown below inside the table)

| Organ | Endpoint | *Fx* | LET (keV *μ*m−1) | RBE |
| --- | --- | --- | --- | --- |
| Spinal cord | Radiation-induced myelopathy | 1 | 10 | a |
|  |  |  | 80 | b |
|  |  | 4 | 10 | c |
|  |  |  | 80 | d |
| more OAR | define your Endpoint | more Fractions | by your decision | your calculation value |

Line 428 measurements with the PinPoint chamber and with a commercial 2D ionization chamber array (OCTAVIUS Detector 1500 XDR, PTW-Freiburg GmbH, Freiburg, Germany)

Where is the location of measurement point and the calibration factor to get the absolute dose of your OCTAVIUS 2D ionization chamber array?

Line 434 We used the point dose measured with the PinPoint chamber to normalize the point dose at the center of the 2D ionization chamber array.

Again, please address now that you have the absolute dose at the center of your OCTAVIUS 2D array, why need to normalize the point dose at the center of the 2D ionization chamber array by using PinPoint chamber? And before normalization, what’s the deviation between these two devices?

Line 495 The TPS-calculated IDDs

Please address how did you convert the Ionization Depth Dose to Integral Depth Dose in carbon ion beam measurement?

Line 512 **Fig 4. Lateral dose profiles in water at selected energies and different positions.**

Please extend the lateral profile range to its lowest potion in Fig. 4

Line 543 Figs 7 (a)–(c)

Please explain why the deviations between the TPS-calculated and the measured dose were always from positive to negative when the field size opens gradually?

Line 548 **Fig 7. Comparison between the calculated and measured absolute point dose at the** **center of the field**

Please make the comparison **of the absolute point dose at the** **center of the field** for the same SOBP with the same carbon energy but **the** **center of the SOBP** locates at different depths.

563 **Fig 8. Comparison of the calculated and measured dose profiles for a field size of 100 mm × 100 mm with a 6 mm SOBP.**

Please describe the solution for the energy-related effective point of measurement of your chamber used for the Ionization depth dose measurement. In other words, how did you correct the Bragg-peak upstream precise position caused by the effective point of measurement in your chamber at different carbon ion energy?

Line 569 **Validation in heterogeneous media**

Again, the measurement here in Fig. 9 is at best to proof the calculation perturbed by the heterogeneous behind the beam central axis, please describe how did your team schedule to do the dose profile along the IDDs?

Line 635 **Fig 11. Patient-specific QA performed in our institute**

The image quality of Fig. 11 is too poor to read, besides, please show the dose profile of X and Y axis but not merely the gamma test pictures.

Line 595 materials was

Correct to; were

Line 635 **Fig 11. Patient-specific QA performed**

**Correct to: was performed**

Line 651 the accuracy by comparing the results to other curve calibration methods and to

It appears that the preposition **to** is redundant. Consider removing it.

Line 658 different curve

Correct to: curves

Line 659 object size. Compared with the polybinary tissue model, our calibration curve showed a difference

Correct to: the difference

Line 666 estimated from the calibrated curve and from

It appears that the preposition **from** is redundant. Consider removing it.

Line 682 depth direction—which increase as the carbon-ion energy increases due to range

The plural verb **increase** does not appear to agree with the singular subject **682 depth direction**. Consider changing the verb form for subject-verb agreement.

Correct to: increases, and to the range

Line 691 measurements was

Correct to: were

Line 697 close to the beam center was in good agreement,

It appears that you have an unnecessary comma before the dependent clause marker **because**. Consider removing the comma.

Line 698 profile were good,

Correct to: profiles

Line 703 influence the TPS-calculated

It seems that **influence** may not agree in number with other words in this phrase.

Correct to: influences

Line 705 considered to compensate

The verb **compensate** is usually in the gerund form when following the word **considered**. Consider replacing it with the **-ing** form.

Correct to: compensating

Line 711 acceptable for the case of superimposed spots in water,

Correct to: in the water,

From line 771 to line 776 However, the MK model, which is a reformulation of the mixed beam model that incorporates the dose dependence of the RBE, has begun to be used in clinical treatments using carbon-ion radiotherapy [9]. Using the parameters of the commissioned beam, we performed an additional analysis to compare the clinical dose distributions on the basis of the mixed beam to that obtained from the MK model, utilizing a prototype VQA Plan that implemented the MK model.

Please clarify the parameters in your institute’s VQA planning system modified of MKM. Please provide the parameters below you use in your institute.

*β*= ? Gy*^−^*^2^, α_ref_ = ? Gy*^−^*^1^, *Rn* = ? *μ*m, and *rd* = ? *μ*m, *z(*)*1D?, *f*1(*z*)?

The factor to compensate the dilution *in vivo* conducted by human circulation scaling factor of your 1.46, is this value from biological effect of a tumor optimized at 10% of HSG (Human Salivary Gland) cell survival *in vitro* or by some other way?

The original Microdosimetric Kinetic Model (MKM) developed by NIRS in 2011. In their model, they separated cell survival curves into α_LET_D+βD^2^, and then use a fixed value of *β*= 0.0615 Gy*^−^*^2^, α_ref_ = 0.12 Gy*^−^*^1^, *Rn* = 3.7 *μ*m, and *rd* = 0.39 *μ*m. The values of *Rn*, *rd* , α0, and β have to be specified and if these parameters are fixed. The beam quality, *z(*)*1D, is solely determined by the distribution f1(*z*) of the microscopic quantity *z*. (Inaniwa *et al* 2015). The value *f*1(*z*), which can be measured by a tissue-equivalent proportional counter (TEPC), with *z^(*)^*1D denoting the saturation-corrected dose mean specific energy of the domain produced by a single event.

The whole items and calculation procedures for cells surviving vs carbon ion beam dose are listed below.

The average number of lethal events of MKM is

with *z(*)*1D denoting the saturation-corrected dose mean specific energy of the domain produced by a single event,

the values of *Rn*, *rd* , α0, and β have to be specified and if these parameters are fixed, the beam quality, *z(*)*1D, is solely determined by the distribution *f*1(*z*) of the microscopic quantity *z*.

*f*1(*z*), which can be measured by a tissue-equivalent proportional counter (TEPC).

The input parameters of the MKM were determined for human salivary gland (HSG) cells using a fixed value of *β*= 0.0615 Gy*^−^*^2^, which has been measured in 200 kVp x-rays.

The values of the other parameter were determined by fitting the predicted doses corresponding to **10% HSG cell survival** at different beam qualities.

As a result, *Rn* = 3.7 *μ*m, *rd* = 0.39 *μ*m and α_0_ = 0.12 Gy*^−^*^1^ were obtained

Once $\bar{N(D)}$ is calculated, then RBE can be calculated logically.

*D*_ref_ refers to the dose of the reference beam quality

For the clinical introduction of the MKM, it was therefore decided to replace the photon reference radiation by a carbon ion reference radiation of a specified beam quality. This beam quality was selected as that at the center of a 6 cm SOBP produced by 350 MeV u−1 carbon ions (reference SOBP). To realize this approach, the biological dose was defined as:

Dclin(x) = scaling factor × Physics Dose x RBE(x).

Dclin(x) denotes clinical prescription dose in carbon ion beam at any certain point x in unit of GyE.

Line 716 sub-beams whenever a lateral

Deleted a and the space in front of lateral

Line 740 a ray casting model, which is one of the improved pencil beam model

Correct to: models

Line 751 and body tissues increases

Correct to: increase

Line 775 dose distributions on the basis of

Correct the on the basis of to: based on

Line 787 models shown

It seems that you are missing a verb. Consider adding it.

Correct to: are shown

Line 792 These patient-specific QA results are clinically acceptable,

It appears that you have an unnecessary comma before the dependent clause marker **because**. Consider removing the comma.
