# Supplementary material for: Commissioning a newly developed treatment planning system, VQA Plan, for fast-raster scanning of carbon-ion beams
Source: PLoS One. 2022 May 10;17(5):e0268087. doi: 10.1371/journal.pone.0268087 (PMC9089877; doi:10.1371/journal.pone.0268087)
Supplement: Attachment — Submitted filename: File_MABLDJ-1_CP_AE-1_MI-5_final.docx [file pone.0268087.s004.docx]

**Review comments of PONE-D-21-22235**

**Commissioning a newly developed treatment planning system, VQA Plan, for fast raster scanning of carbon-ion beams**

**General comments:**

The readability is acceptable in general with minimal grammar errors. It does come across a little dense at times. Although the manuscript is reasonably well readable, the English could be improved.

1. Paper structure and presentation are too lengthy in the current version and need to be more concise.

-> Please read another document for our answer to this question.

2. Tables and figures, especially the quality of all figures, need to be improved in the revision.

-> Please read another document for our answer to this question.

3. Language also needs to be improved by native English speakers. Please consult the reviewers' comments for more information.

-> Please read another document for our answer to this question.

The manuscript is lacking of the core value introduction of VQA which is a flaw that detracts from the positive contributions of this article. The author is encouraged to address more superiority of their planning system VQA.

-> Please read another document for our answer to this question.

Specific comments:

Line 48 accuracy for heterogeneous media

The singular noun **accuracy** follows a number other than *one*. Consider changing the noun to the plural form. Correct to: accuracies

-> Thank you for your comment. Accordingly, we have revised “accuracy” to “accuracies”.

Line111 optimized for each individual

**Each individual** may be redundant.

-> Thank you for your comment. The text has been amended to “each clinical case”.

Line114 gland (HSG) tumor cells has been

The singular verb **has** does not appear to agree with the plural subject **cells**. Consider changing the verb form for subject-verb agreement.

-> Thank you for your comment. In this line,“survival” is the subject and the verb should be singular.

Line 118 beams, and the response to individual tumors is considered on the basis of the…

It appears that you have an unnecessary comma in a compound subject. Consider removing it.

-> As recommended, we have removed the comma.

The verb **is** does not seem to agree with the subject. Consider changing the verb form. Correction: Is to are

-> Thank you for your comment. As recommended, we have changed “it” to “are”.

The phrase **on the basis of** may be wordy. Consider changing the wording. Correction: Based on

-> Thank you for your comment. Accordingly, we have changed “on the basis of” to “based on”.

Line 130 provide 100 different energy selections between 73.3 and 430.0 MeV/u from 12 accelerated energies,

Please clarify 100 different energies and 12 accelerated energies, what is the difference between these two energies.

-> Thank you for your comment. We have inserted the word “nominal” to distinguish the energies obtained by raster scanning from the accelerated energies.

Line 137 The main dose monitor checks the dose remaining at the given position, and a spot position monitor (SPM) confirms the position of the spot.

Does this specify for one carbon energy? or for every energy? Please add more explanations.

-> Thank you for your comment. The correct term is “for every energy”. The text has been modified accordingly.

Line 138 position, and a spot position monitor (SPM) confirms the position of the spot. A monitor

It appears that you have an unnecessary comma in a compound subject. Consider removing it.

-> Thank you for your comment. We have removed the comma as suggested.

Line 139 unit (MU) is defined on the basis of a fixed amount of charge collected in the ionization

The phrase **on the basis of** may be wordy. Consider changing the wording. Correction: Based on

-> Thank you for your comment. Accordingly, we have changed “on the basis of” to “based on”.

Line 140 chamber of the main dose monitor, and it corresponds to a physical dose to water of

Correction: the water

-> Thank you for your comment. We have made the suggested change to “ the water”.

Line 153 The input data required for the scanned carbon-ion beam are integral depth doses (IDDs), the in-air lateral profiles, measurements relating to fragmentation, and the absolute dose and absolute dose correction factor.

Please add more explanations on what type of ion chamber you used for (IDDs), in-air lateral profiles as well as the data with a range shifter (RS) measurements.

-> Thank you for your comment. The revised manuscript states the type of ion chamber used in each measurement.

Line163 measurement data are described in [2], so only a short summary

**Short summary** may be redundant. Correction: removed

-> Thank you for your comment. As recommended, we have removed the phrase “so only a short summary...”

Line 173 , (1)

It appears that you have improperly spaced some punctuation. Consider removing a space.

-> Thank you for your comment. As suggested, we have removed the extra space.

Line 180 first component (*n* = 1) represents the incident carbon-ion beam,

It appears that you have an unnecessary comma in a compound object. Consider removing it.

-> Thank you for your comment. We have removed the comma in the revised text.

Line 199 multiplying the beam current defined at every control point by the time period.

**Time period** may be redundant. Keep only one word.

-> Thank you for your comment. Accordingly, we have revised “time period” to “period”.

Line 200-203 Please address how to deal with the transit dose from one spot to the other spot in Intensive Modulated Carbon Therapy of your raster scanning facility.

-> The transit dose in Intensive Modulated Carbon Therapy is handled by the same method as the one used in the single-field uniform dose (SFUD). The transit dose depends on the beam intensity. The text has been modified accordingly.

Line 203 of the dose needed to calculate the beam width in the SPM is less than 0.00018 MU

What is the precise control mechanism for such a low MU?

-> The SPM uses a multi wire proportional chamber (MWPC) filled with 90% Ar and 10% CO_2_. The MWPC runs the electron avalanche amplification that increases the measurement precision of low doses.

Line 204 5) if the ratio of the stopping dose and the moving dose is not 1:1.

Please define what are: stopping dose and the moving dose?

-> The text has been amended as follows: “The ratio of the stopping dose (the deposited dose at a given point) and the moving (transit) dose is not 1:1”.

Line 212 photons and for carbon ions using a specified survival level (10%)

Please clarify what is the specified survival level (10%)? Is that HSG? Following the same question, we all realize there are only one normal issue cell line (such as HSG) adopted by using a cell survival model to predict the carbon ion RBE, did your institute ever try to use individual tumor cell line for predicting the corresponding RBE to achieve disease by disease precision treatment in carbon ion beam treatment? Despite the difficult to do individual tumor cell line for predicting the RBE to achieve disease by disease precision treatment, what are your comments for this strategy?

-> Thank you for identifying this omission. The specified survival level (10%) is HSG. We have clarified this point by adding “based on the LQM with HSG” in the revised manuscript. HSG was found being an averaged radiosensitivity compared to other cell lines. The following paper reported the observation.

Kanai T, Endo M, Minohara S, Miyahara N, Koyama-Ito H, Tomura H, et al. Biophysical characteristics of HIMAC clinical irradiation system for heavy-ion radiation therapy. International Journal of Radiation Oncology* Biology* Physics. 1999;44(1):201-10.

Line 214 , (3)

It appears that you have improperly spaced some punctuation. Consider removing a space.

-> Thank you for your comment. We have removed the additional space.

Line 225 , (5)

It appears that you have improperly spaced some punctuation. Consider removing a space.

-> Thank you for your comment. We have removed the additional space.

Line 226 , (6)

It appears that you have improperly spaced some punctuation. Consider removing a space.

-> Thank you for your comment. We have removed the additional space.

Line 237 contributions from the carbon ions and from

It appears that the preposition **from** is redundant. Consider removing it.

-> Thank you for your comment. The revised sentence reads “contributions received from the carbon and fragment isotopes”.

Line 238 Gaussian form, the first component (*n* = 1) represents the carbon isotopes,

It appears that you have an unnecessary comma in a compound object. Consider removing it.

-> Thank you for your comment. We have removed the unnecessary comma from this sentence.

Line 254 where *dclin,i* denotes the clinical dose at voxel *i*. The scaling factor 1.46

It seems that preposition use may be incorrect here. It is to be: of 1.46.

-> Thank you for your comment. We have amended the phrase to “The scaling factor was 1.46”.

The author declares the scaling factor 1.46 was determined by connecting the modeled RBE to the clinical experience with neutron therapy in the report of Biophysical characteristics of HIMAC clinical irradiation system for heavy-ion radiation therapy, please explain or upon your opinion why use neutron to define carbon heavy ion scaling factor since neutron is neutral particle while carbon ion used for radiation therapy is charged particle.

-> Neutrons are neutral particles but their interactions generate charged particles such as recoil protons. In HIMAC, clinical experience of neutron therapy had been acquired before starting carbon-ion therapy. For instance, the dose amount (prescription dose) in cancer treatment by the neutron beam was known. The biological RBE of the neutron beam was equivalent to one of the carbon ion at 80 keV/μm. To determine the prescription dose of carbon-ion therapy RBE of carbon ions at 80 keV/μm was scaled to match the clinical RBE of the neutrons (i.e., 3). The scaling factor is 1.46.

Line 264 parameters needed

It seems that you are missing a verb. Consider adding it. It is to be: are needed

-> Thank you for your comment. The phrase has been recast as “parameters that describe...”

Line 270 of the absolute doses to convert the IDDs to absolute values.

Please describe how you define the absolute doses? Where to do the measurement along the IDDs in different carbon ion energies?

-> The absolute dose is defined at 20 mm from the surface except at 100 and 140 MeV/u, where it is defined at 2 and 5 mm, respectively. The explanation has been provided in line 350 of the revised text.

Line 270 of the absolute doses to convert the IDDs to absolute values. The absolute correction factor is used to compensate for the difference between the calculated physical absolute dose and that obtained from the measurements.

What is the absolute correction factor? Please clarify the definition and base on what to compensate the difference of physical absolute dose between calculation and measurements?

-> The absolute correction factor is derived from the analysis result of IDNF. This factor corrects the dose difference between the measurement and the dose calculated using the modeled beam. The explanation is given in line 705 of the revised text.

Line 274 **Determination of the CT-number-to-relative-stopping-power curve, validation,** **and range error estimation**

In this section, what is the abbreviation of RSP?

-> The term RSP stands for relative-stopping power. It is defined when first used on line 103.

Please provide your methods and experiences on how to deal with a patient with artifacts occurred by I-125 seeds implant, or by other metal materials which the CT number exceeds your CT-number-to-RSP calibration table, and the dose calculation accuracy under these artifacts.

-> Investigating the dose calculation accuracy under artifacts from a high-density material is beyond the scope of this manuscript. In clinical practice, a high-density material is replaced with an appropriate material. We also avoid the use of a beam that penetrates the high-density material. Artifacts on the CT image are suppressed using a metal-artifact reduction algorithm.

For those patients who have metal materials inside or beside the lesion, will you gona treat by heavy ion? If yes, what is your dose confidence? If not for treatment, what is your next strategy for patients?

-> For patients having metal materials inside or beside the lesion, we process CT images using the metal-artifact reduction technique and apply a beam that does not penetrate the metal. Under these conditions, the dose calculation accuracy should be the accuracy reported in our manuscript. If the patient cannot be treated via carbon-ion therapy, the next strategy will be indicated by a radiation oncologist.

In **TPS validation** section in line 359 We created all the fields by superimposing pencil beams. We obtained measurements with the Advanced Markus chamber, except that we used the PinPoint chamber for field sizes less than 40 mm × 40 mm

According to the author’s description, for all clinical data implemented to your VQA TP system were measured by Advanced Markus chamber except the use of PinPoint chamber for field sizes less than 40 mm × 40 mm. The reviewer would like to ask how did you do the effective point of measurement correction of these two chambers?

-> As an effective point, we used the geometric center of the PinPoint chamber and the surface of the Advanced Markus chamber.

Line 289 eV [21], and the particle velocity relative to the speed of light in vacuum

The noun phrase **vacuum** seems to be missing a determiner before it. Consider adding an article.

Correction: in a vacuum

-> Thank you for your comment. The phrase has been changed to “in a vacuum” in the revised text.

Line 305 the relationship between CT number and RSP by means of

The phrase **by means of** may be wordy. Consider changing the wording.

Consider to use: using, utilizing, employing, through

-> Thank you for the comment. I have replaced “by means of” with “using” in the revised text.

Line 307 We conducted an experiment

The phrase **conducted an experiment** may be unnecessarily wordy. Consider replacing the noun with a corresponding verb.

Consider to use: experimented

-> Thank you for your comment. I have replaced “conducted an experiment” with “experimented” in the revised text.

Line 318 method can be found in [25, 26]. In short, uncertainties in patient

Correct to: in patient’s

-> Thank you for the comment. I have corrected the word to “patient’s”.

Line 333 mm, using the StingRay

Please clarify the term StingRay.

-> Thank you for your comment. We have introduced StingRay in line 153.

Line 335 IDDs on the dose–area product (DAP) [27] obtained

Correct to: were obtained

-> As requested, I have amended the phrase to “were obtained...”

Line 372 The calculation grid is fixed at 2.0 mm × 2.0 mm × 2.0 mm and is not adjustable by the user.

What if you have to treat a small lesion less than 2 mm like an eye ball melanoma then you restrict the planner changing the resolution for better dosimetric evaluation inside the lesion and the surrounding normal critical tissues/organs?

-> We appreciate your question. At coarse resolution, we would underestimate the dose to the critical organs because of the volume averaging effect.

Line 375 **Validation in heterogeneous media**

The author declares to place lung- and bone-equivalent materials 40 mm upstream from the surface of the water phantom to investigate the heterogeneous media, well, it seems at best to investigate the attenuation of the materials interested, please try to use Gafchromic film or of your own way for the measurement of dose profile inside the heterogeneous materials, especially the detail measurement of beam profile at the entrance and exit dose along the heterogeneous material.

-> We are conducting a detailed study to characterize the dose calculation accuracy in the VQA Plan for heterogeneous media. The suggested measurement will be included in the study and separately reported.

Line 387 We measured the RSP for each material comprising the phantom in the same manner as in the experiment used to validate the CT-number-to-RSP curve, and this was reflected in the calculation by overwriting the CT-number function after inversely estimating the CT number of the measured RSP in the created

CT-number-to-RSP table because the material comprising the phantom was not real tissue and the CT number acquired from the CT image was not as accurate as that of the tissue.

Please re-write these sentences item by item to avoid confused. At this moment, reader couldn’t catch what you try to express.

-> Thank you for your comment. We have rewritten the sentences item by item as recommended. Please see line 386.

Line 377 150 mm × 150 mm × 20 mm of acrylic and 30 mm × 30 mm × 20 mm of lung-

Correct to: of the lung-

-> Thank you for the comment. The phrase has been corrected to “of the lung”.

Line 378 bone-equivalent materials was

Correct to: were

-> Thank you for the comment. The phrase has been corrected to “bone-equivalent materials were...”

Line 382 point doses along the central axis and measure

Consider changing the verb form for subject-verb agreement.

Correct to: measures

-> Thank you for your comment. We suppose the singular “measure” is correct in this sentence because it is infinitive verb.

Line 392 tissue and the CT number acquired from the CT image was not as accurate as that

Correct to: as of that

-> Thank you for your comment. I have corrected the phrase to “not as accurate as that of the tissue”.

Line 399 TPS using the RBE measurements reported in [2],

Please add Reference in front of [2], and correct line 844 Therapy Center. J Appl Clin Med Phys. Forthcoming. Forthcoming to: Article-in-press

-> Thank you for identifying these errors. The reference has been published and we have corrected it in the revised text. Please see line 396.

Line 442 from six head and neck cancer cases treated in the OHITC. In the prostate

Correct to: In prostate

-> Thank you for your comment. The phrase has been amended to “in prostate” in the revised manuscript.

Line 446 **Results**

All figures are in very poor image qualities. All figures should be retouched and gallery proofed in the next version.

-> We appreciate your comment. We had asked a journal officer about the quality of the figures because our submitted figures met the journal criteria. The officer replied that the submission PDF is for reviewing purposes only and does not reflect the appearance of the final publication.

The officer also mentioned that editors and reviewers will have access to the original source files via the blue download link on the top right corner of the PDF rendering of our figure.

Line 448 Fig 1 (a) shows the determined CT-number-to-RSP curve. The solid line is for small-diameter cases, such as the head and neck region, and the dashed line is for large-diameter cases, such as the abdominal region.

Please explain the reason what cause the RSP of the body lower than that of the head at a higher CT numbers?

-> The phantom size causes the beam-hardening effect, which induces a change in CT number of the same tissue and an RSP difference at the same CT number.

Line 467 Figs 1 (d) and (e) the results of our experimental verifications.

Add showed after (e)

- Thank you for pointing out this error. The phrase has been corrected to “Figs. 1 (d) and 1 (e) show the results of our experimental verifications”.

Line 469 differences between the RSP determined from the calibrated curves and from

It appears that the preposition **from** is redundant. Consider removing it.

-> Thank you for your comment. I have removed the second instance of “from” in this sentence.

Line 472 tissue,

It appears that you have an unnecessary comma in a compound subject. Consider removing it.

-> Thank you for the comment. The unnecessary comma has been removed.

Line 503 **Fig 3. Comparison of IDDs from the TPS calculations and from**

It appears that the preposition **from** is redundant. Consider removing it.

-> Thank you for your comment. I have removed the second instance of “from” in the figure caption.

**Fig 3. Comparison of IDDs from the TPS calculations and from measurement for all 100 energies.** The circles show the measured IDDs and the solid lines represent the calculated IDDs. The lower graph shows the differences in R90 in units of millimeters for all energies.

The author should present no more than 10 IDDs between the smallest and the largest carbon ion energies to avoid the crowded and fussy noise in Fig. 3.

-> Thank you for indicating the clutter in our original Figure 3. The IDDs have been replotted with intervals of approximately 10 MeV/u.

Line 512 **Fig 4. Lateral dose profiles in water at selected energies and different positions.**

It is difficult to read each figure’s legendary, please correct. Please show Fig. 4 each profile’s tails both at the right and left side to a reasonable margin.

-> Thank you for your comment. We had asked a journal officer about the quality of the figures because our submitted figures met the journal criteria and the legend was readable. The officer replied that the submission PDF is for reviewing purposes only and does not reflect the appearance of the final publication.

The officer also mentioned that editors and reviewers will have access to the original source files via the blue download link on the top right corner of the PDF rendering of our figure.

Besides, what if the scanning beam passes over the desired margin, is there any erroneous proof mechanism to avoid scanning beam out of control?

-> The beam spot is monitored using a spot position monitor (SPM). The SPM has a tolerance of deviation from the planned position. If a beam exceeds the tolerance, it is stopped.

Line 522 **Fig 5. Comparison of the absolute doses from the TPS and from**

It appears that the preposition **from** is redundant. Consider removing it.

-> Thank you for your comment. I have removed the second instance of “from” in the figure caption.

What is the corresponding RBE in the same LET but different fraction size in your VQA TPS? Please tabulate the results for representative carbon ion energies. For example, please make the comparison for different OARs(skin, Spinal cord, kidney, liver after partial heaptectomy, Intestine, et al) with the endpoint by different fractions in the fix LET to get the RBE ( like as a, b, c, d are the desired items shown below inside the table)

-> The RBE does not depend on the fraction number. We cannot fill out the table.

| Organ | Endpoint | *Fx* | LET (keV *μ*m−1) | RBE |
| --- | --- | --- | --- | --- |
| Spinal cord | Radiation-induced myelopathy | 1 | 10 | a |
|  |  |  | 80 | b |
|  |  | 4 | 10 | c |
|  |  |  | 80 | d |
| more OAR | define your Endpoint | more Fractions | by your decision | your calculation value |

Line 428 measurements with the PinPoint chamber and with a commercial 2D ionization chamber array (OCTAVIUS Detector 1500 XDR, PTW-Freiburg GmbH, Freiburg, Germany)

Where is the location of measurement point and the calibration factor to get the absolute dose of your OCTAVIUS 2D ionization chamber array?

->. We irradiated 10 cm × 10 cm fields at three different energies and averaged the measured values to get the absolute dose over the 2D array.

Line 434 We used the point dose measured with the PinPoint chamber to normalize the point dose at the center of the 2D ionization chamber array.

Again, please address now that you have the absolute dose at the center of your OCTAVIUS 2D array, why need to normalize the point dose at the center of the 2D ionization chamber array by using PinPoint chamber? And before normalization, what’s the deviation between these two devices?

-> We realized that lengthening the time irradiation of the 2D array caused a systematic dose error. The volume averaging effect also entered the measurement. Then, we developed a correction method to overcome the systematic error.

Line 495 The TPS-calculated IDDs

Please address how did you convert the Ionization Depth Dose to Integral Depth Dose in carbon ion beam measurement?

-> The beam in the Monte Carlo simulation was modeled using the measurements obtained in the StingRay chamber. The Monte Carlo simulation estimated particles that could not be measured in the StingRay chamber, such as large-angle-scattered particles that escaped the limits of the chamber size. Finally, the integral depth dose was derived from the Monte Carlo simulation.

Line 512 **Fig 4. Lateral dose profiles in water at selected energies and different positions.**

Please extend the lateral profile range to its lowest potion in Fig. 4

-> The value “1” on the log scale on the vertical axis converts to “0” on the linear scale. This is the lowest portion.

Line 543 Figs 7 (a)–(c)

Please explain why the deviations between the TPS-calculated and the measured dose were always from positive to negative when the field size opens gradually?

-> We assumed that the contribution to the dose from the fragments is larger in the measurement than in the modeled beam.

Line 548 **Fig 7. Comparison between the calculated and measured absolute point dose at the** **center of the field**

Please make the comparison **of the absolute point dose at the** **center of the field** for the same SOBP with the same carbon energy but **the** **center of the SOBP** locates at different depths.

-> We appreciate your comment. Figure 7 indicates the absolute point dose at the center of the field for the same SOBP with the same carbon energy; however, the center of the SOBP locates at different depths.

563 **Fig 8. Comparison of the calculated and measured dose profiles for a field size of 100 mm × 100 mm with a 6 mm SOBP.**

Please describe the solution for the energy-related effective point of measurement of your chamber used for the Ionization depth dose measurement. In other words, how did you correct the Bragg-peak upstream precise position caused by the effective point of measurement in your chamber at different carbon ion energy?

-> We appreciate your inquiry. There is no energy-related effective point for the carbon-ion beam.

Line 569 **Validation in heterogeneous media**

Again, the measurement here in Fig. 9 is at best to proof the calculation perturbed by the heterogeneous behind the beam central axis, please describe how did your team schedule to do the dose profile along the IDDs?

-> We decided the measurement depths of the dose profile after measuring the IDD. We could then identify the depth at which the dose profile was rapidly changing.

Line 635 **Fig 11. Patient-specific QA performed in our institute**

The image quality of Fig. 11 is too poor to read, besides, please show the dose profile of X and Y axis but not merely the gamma test pictures.

-> Thank you for the comment. We have added the dose profile. Regarding the quality of the figures, please see our responses to earlier queries. Lines 446 and 512.

Line 595 materials was

Correct to; were

-> Thank you for your comment. The phrase has been corrected to “materials were...”

Line 635 **Fig 11. Patient-specific QA performed**

**Correct to: was performed**

-> Thank you for your comment. The phrase has been corrected to “Patient-specific QA was performed…”

Line 651 the accuracy by comparing the results to other curve calibration methods and to

It appears that the preposition **to** is redundant. Consider removing it.

Thank you for your comment. I have removed the redundant word “to”.

Line 658 different curve

Correct to: curves

-> Thank you for pointing out this error. It has been amended to “different curves”.

Line 659 object size. Compared with the polybinary tissue model, our calibration curve showed a difference

Correct to: the difference

-> Thank you for the comment. We suppose indefinite article is correct in this sentence.

Line 666 estimated from the calibrated curve and from

It appears that the preposition **from** is redundant. Consider removing it.

-> Thank you for your comment. I have removed the second instance of “from” in this sentence.

Line 682 depth direction—which increase as the carbon-ion energy increases due to range

The plural verb **increase** does not appear to agree with the singular subject **682 depth direction**. Consider changing the verb form for subject-verb agreement.

Correct to: increases, and to the range

-> Thank you for your comment. The phrase has been corrected to “which increases as the carbon-ion energy increases…” in the revised text.

Line 691 measurements was

Correct to: were

-> Thank you for your comment. We have amended “was” to “were”.

Line 697 close to the beam center was in good agreement,

It appears that you have an unnecessary comma before the dependent clause marker **because**. Consider removing the comma.

-> Thank you for your comment. We have removed the redundant comma.

Line 698 profile were good,

Correct to: profiles

-> Thank you for your comment. The phrase “profile were good” has been corrected to “profiles were good”.

Line 703 influence the TPS-calculated

It seems that **influence** may not agree in number with other words in this phrase.

Correct to: influences

-> Thank you for your comment. The subject is plural and the verb is correct.

Line 705 considered to compensate

The verb **compensate** is usually in the gerund form when following the word **considered**. Consider replacing it with the **-ing** form.

Correct to: compensating

-> We appreciate your comment. The original term “compensate” is correct.

Line 711 acceptable for the case of superimposed spots in water,

Correct to: in the water,

-> Thank you for your comment. The phrase has been amended to “superimposed spots in the water”.

From line 771 to line 776 However, the MK model, which is a reformulation of the mixed beam model that incorporates the dose dependence of the RBE, has begun to be used in clinical treatments using carbon-ion radiotherapy [9]. Using the parameters of the commissioned beam, we performed an additional analysis to compare the clinical dose distributions on the basis of the mixed beam to that obtained from the MK model, utilizing a prototype VQA Plan that implemented the MK model.

Please clarify the parameters in your institute’s VQA planning system modified of MKM. Please provide the parameters below you use in your institute.

*β*= ? Gy*^−^*^2^, α_ref_ = ? Gy*^−^*^1^, *Rn* = ? *μ*m, and *rd* = ? *μ*m, *z(*)*1D?, *f*1(*z*)?

-> We took reference values of β = 0.0615 Gy*^−^*^2^, α_ref_ = 0.796 Gy*^−^*^1^, Rn = 3.9 μm, and Rd = 0.32 μm. z(*)1D is defined in Equation 3 in the reference and f1(z) is determined during the calculation of Equation 7 in the reference.

Taku Inaniwa et al 2010 Phys. Med. Biol. 55 6721

The factor to compensate the dilution *in vivo* conducted by human circulation scaling factor of your 1.46, is this value from biological effect of a tumor optimized at 10% of HSG (Human Salivary Gland) cell survival *in vitro* or by some other way?

->In HIMAC, the clinical experience of neutron therapy had been acquired before staring carbon-ion therapy. For instance, the dose amount (prescription dose) in cancer treatment with the neutron beam was known. The biological RBE of the neutron was equivalent to one carbon ion at 80 keV/um. To determine the prescription dose of carbon-ion therapy, the biological RBE of carbon ions at 80 keV/um was scaled to match the clinical RBE of the neutrons (i.e., 3). The scaling factor is 1.46.

The original Microdosimetric Kinetic Model (MKM) developed by NIRS in 2011. In their model, they separated cell survival curves into α_LET_D+βD^2^, and then use a fixed value of *β*= 0.0615 Gy*^−^*^2^, α_ref_ = 0.12 Gy*^−^*^1^, *Rn* = 3.7 *μ*m, and *rd* = 0.39 *μ*m. The values of *Rn*, *rd* , α0, and β have to be specified and if these parameters are fixed. The beam quality, *z(*)*1D, is solely determined by the distribution f1(*z*) of the microscopic quantity *z*. (Inaniwa *et al* 2015). The value *f*1(*z*), which can be measured by a tissue-equivalent proportional counter (TEPC), with *z^(*)^*1D denoting the saturation-corrected dose mean specific energy of the domain produced by a single event.

The whole items and calculation procedures for cells surviving vs carbon ion beam dose are listed below.

The average number of lethal events of MKM is

with *z(*)*1D denoting the saturation-corrected dose mean specific energy of the domain produced by a single event,

the values of *Rn*, *rd* , α0, and β have to be specified and if these parameters are fixed, the beam quality, *z(*)*1D, is solely determined by the distribution *f*1(*z*) of the microscopic quantity *z*.

*f*1(*z*), which can be measured by a tissue-equivalent proportional counter (TEPC).

The input parameters of the MKM were determined for human salivary gland (HSG) cells using a fixed value of *β*= 0.0615 Gy*^−^*^2^, which has been measured in 200 kVp x-rays.

The values of the other parameter were determined by fitting the predicted doses corresponding to **10% HSG cell survival** at different beam qualities.

As a result, *Rn* = 3.7 *μ*m, *rd* = 0.39 *μ*m and α_0_ = 0.12 Gy*^−^*^1^ were obtained

Once $\bar{N(D)}$ is calculated, then RBE can be calculated logically.

*D*_ref_ refers to the dose of the reference beam quality

For the clinical introduction of the MKM, it was therefore decided to replace the photon reference radiation by a carbon ion reference radiation of a specified beam quality. This beam quality was selected as that at the center of a 6 cm SOBP produced by 350 MeV u−1 carbon ions (reference SOBP). To realize this approach, the biological dose was defined as:

Dclin(x) = scaling factor × Physics Dose x RBE(x).

Dclin(x) denotes clinical prescription dose in carbon ion beam at any certain point x in unit of GyE.

Line 716 sub-beams whenever a lateral

Deleted a and the space in front of lateral

-> Thank you for your comment. We have corrected the phrase to “sub-beams whenever lateral...”

Line 740 a ray casting model, which is one of the improved pencil beam model

Correct to: models

-> Thank you for your comment. The phrase “one of the improved pencil beam model” has been revised to “one of the improved pencil beam models” in the revised text.

Line 751 and body tissues increases

Correct to: increase

-> Thank you for your comment. The phrase “and body tissues increases” has been amended to “and body tissues increase…”

Line 775 dose distributions on the basis of

Correct the on the basis of to: based on

-> As recommended, “on the basis of” has been revised as “based on”.

Line 787 models shown

It seems that you are missing a verb. Consider adding it.

Correct to: are shown

-> Thank you for your comment. The phrase has been corrected to “models are shown”.

Line 792 These patient-specific QA results are clinically acceptable,

It appears that you have an unnecessary comma before the dependent clause marker **because**. Consider removing the comma.

-> Thank you for your comment. We have removed the redundant comma.
